# Factors influencing adherence to antiretroviral treatment among adults accessing care from private health facilities in Malawi

**DOI:** 10.1186/s12889-019-7768-z

**Published:** 2019-10-28

**Authors:** Lusungu Chirambo, Martha Valeta, Tifiness Mary Banda Kamanga, Alinane Linda Nyondo-Mipando

**Affiliations:** 1 Department of Public Health, School of Public Health and Family Medicine, College of Medicine, Private Bag 360, Blantyre, Malawi; 2 Department of Health Systems and Policy, School of Public Health and Family Medicine, College of Medicine, Blantyre, Malawi

## Abstract

**Background:**

Private health facilities are increasingly being recognized as the neglected partner in the provision of HIV services. The non-adherence rate in the study sites ranged from 19 to 22%. This study explored the factors associated with non-adherence from antiretroviral therapy (ART) among adult patients accessing ART services at two privately owned urban health facilities in Malawi.

**Methods:**

We conducted a descriptive qualitative approach employing in-depth interviews among adults who either defaulted or were retained in HIV care in two privately owned facilities in Malawi from March to July 2017. We purposively selected participants and interviewed a total of 6 ART providers and 24 ART clients. Data were analyzed manually using a thematic approach.

**Results:**

Overall, participants identified four facilitators for retention in care and four broad categories of barriers namely individual, psychological, drug related and human resource related factors. The factors that facilitated retention in care included follow up visits after missing a visit, adequate information education and counseling, and supportive relationships.

**Conclusion:**

The main reason for defaulting from antiretrovirals (ARVs) was fear of disclosing an HIV status to avert potential stigma and discrimination. In implementing ART clinics due consideration and strategies need to be adopted to ensure that privacy and confidentiality is preserved. Although adoption of all the key Malawi Implementing strategies like expert clients and a guardian may optimize retention in care, there is need for prior analysis of how those may lead to unintended disclosure which inadvertently affects adherence. Furthermore, private facilities should orient their clients to the public facilities within the catchment area so that clients have an option for alternative access to HIV care in the event of financial constraints.

## Background

Retention in HIV care remains a major goal for HIV services worldwide. Globally default rates vary from 32.7% in America, 12.1% in Europe to 39.4 to 79.4% in Africa [1]. Malawi has made positive strides with the non-adherence rate that has stabilized at 1.5% overall with variations in specific health facilities [2]. Non-adherence to treatment reduces the immunological benefits of ARVs which predisposes clients to opportunistic infections, increases both the risk of drug resistance and HIV transmission [3].

Patient level factors that contribute to defaulting from ART include forgetfulness [4] fatigue, hopelessness [5], absence of symptoms and severity of the illness [6–8]. Furthermore, lack of support from a partner [6, 7] negative perceptions towards ART medication, pre-occupation [3, 9, 10] and absence from home due to employment [4] compromises adherence to ARVs. Stigma and discrimination coupled with family pressure [5, 11], regular changes of residence [12, 13] and religious beliefs [5] influence defaulting from ART. Financial cost associated with accessing treatment is secondary to long distance especially among those residing in rural areas [3, 11].

Inefficient health system [3, 9, 14] including inadequate counseling on benefits of ART [1], long waiting times [9], compromised privacy due to inadequate consultation rooms [3, 11–16], intermittent supply and stock outs of antiretroviral and reagents [17] and dissatisfaction with the care received [13] contribute to defaulting from ART. Drug related factors that influence defaulting include complexities and side effects of ARV regimen [3, 12, 13]. Further, high pill burden compounds drug related factors [16].

ART services in Malawi are provided by both privately and government-owned health facilities [18, 19]. Private facilities are increasingly being recognized as neglected partner in the provision of HIV services [18]. Patients accessing ART care from private facilities are usually those that can pay for the services out of pocket or through a medical insurance. About 66% of the privately-owned facilities provide at least one HIV-related service with the common service being HIV testing and counseling [20]. Of these, 63% provide testing only while 36% provide both testing and ART treatment [20].

Previous studies on factors associated with ART default have primarily been conducted in government owned health facilities with very limited attention given to privately owned facilities. The current defaulting rate in the two privately owned facilities under study for the period January 2012 to December 2016 averaged 19 to 22% respectively [21, 22] thus higher than national average. This study explored factors associated with ART default among adult patients accessing care at the two privately owned health facilities in the Southern Malawi. We defined ART defaulting as per Malawi HIV guidelines [23] that classifies one as a defaulter when one does not report to the clinic for two consecutive months and is projected to have run out of drugs with no information on whether one stopped neither transferred out nor dead.

The Health Belief Model guided the study because it creates a better framework for studying health behaviour [24]. The tenets of this model informed the topic guide. We conceptualized perceived susceptibility to AIDS in people living wth HIV as a motivating factor to take ARVs to remain virally suppressed. We applied perceived severity in the aspects of the topic guide that asked participants of their health when they were initiating on ARVs and if they knew their immunological status. Perceived barriers formed the greater aspect of the guide by asking various forms of barriers to ART adherence. We interpreted cues to action in this study as any factors that motivated participants to remain on ARVs while self efficacy was covered when we asked participants on their knowledge over their condition and ability care for themselves [24].

## Methods

A descriptive qualitative approach [24] was employed to assess factors influencing defaulting from ART among adults accessing their ARVs at two privately owned facilities in urban settings in the southern part of Malawi. A descriptive qualitative study allowed for a naturalistic inquiry of the phenomena and presentation of a descriptive summary of results [24]. We opted for in-depth interviews because of the depth they guarantee in understanding a social phenomena [25, 26]. In-depth Interviews ensured privacy among the participants cognizant of the sensitive nature of the topic.

### Study setting

The two privately owned health facilities are among the major centers providing health care services in Malawi to the general public at a cost for profit. The facilities provide HIV services, specialized services such as gynecology, obstetrics pediatrics, ophthalmology, dermatology and orthopedics. The staff at the clinics consists of Medical Officers, Clinical Officers, State Registered Nurses, Nurse Midwife Technicians, Pharmacy Technicians, Radiographer Technicians and Dental Technicians. Both clinics attend to outpatients and inpatients and refer cases as necessary to other hospitals. One clinic had a bed capacity of 15 while the other had 11.

HIV services are open to both insured and non-insured clients and are offered daily. Patients using these facilities pay either through medical insurance schemes or paying out-of-pocket after every appointment. Majority of the clients using these clinics are either formally employed or self-employed. Patients that are not under any medical insurance pay a consultation fee of 0.65USD at each visit when they refill their ARVs. Patients attend the ART clinic each month and they are initially provided with ARVs to last them for a month and then quarterly on subsequent visits. At each visit the status of each client is captured on the follow-up cards and in the ART registers. The two facilities have a varied patient volume load with one clinic at 500 ART clients per month while the other attends to 100 clients per month.

### Sample size

We drew a purposive [27] sample of participants from a cohort of patients who initiated ART between the periods of January 2012 to December 2015 and health workers with more than a years’ working experience at each privately owned health facility. A total of 30 participants were recruited in the study. Of these, we purposively selected 12 clients who had defaulted from their ARVs (six males and six females), 12 clients who were compliant to their ARVs (six males and six females). Of the 6 health care workers two were clinical officers, two were nurses that run the ART clinic and two were clerks in the ART clinic. We defined defaulting as per Malawi HIV guidelines [26] that classifies one as a defaulter when one does not report to the clinic for two consecutive months and is projected to have run out of drugs with no information on whether one stopped neither transferred out nor dead. We defined a compliant client as client as one is alive, is on ART and has never stopped taking ART treatment since January 2012 to Dec 2015 (time of data collection). We included multiple characteristics in our sampled participants such as age, gender, residency, education level, marital status, religion, occupation, type of ART regimen, compliance status and duration on ART regimen in order to achieve maximum variation [27, 28] to improve credibility and trustworthiness of the study.

### Sampling

We identified potential participants who met the definition of defaulting and adherent by reviewing the ART registers from the two privately owned health facilities from 1st January 2012 to 31st December 2015. We used this cut-off period because it registered higher default rates (22 and 19% in the two clinics respectively) as compared to the period 2008–2011 when registered rates were 10 and 4% respectively [21, 22]. If the client met the definition of adherent or non-adherent criteria as defined by Malawi HIV Guidelines [23], he or she was listed as a potential participant and a research assistant contacted him/her to ascertain whether they were continuing with ARVs from other clinics or not. If the client was not continuing with ART at any facility, a research assistant asked if they were willing to hear more about the study that was taking place. Upon agreement, an appointment was scheduled at a place convenient to them for further explanation about the study and for their informed consent for participation. The adherent clients were also called and we solicited their interest with study participation. Of all clients approached, four refused to participate citing time constraints and unwillingness as the reasons for non-participation.

### Data collection

We collected data from March to July 2017 using pretested interview guides that were developed based on the study objectives. The Principal Investigator (LC) conducted all the interviews with assistance from two trained research assistants. The research assistants were recruited and trained in ethics and logistics of the study and assisted in the identification of participants at clinic level, scheduling appointments and identifying private venues for the IDIs and KIIs within the facilities. All interviews were face-to-face and were conducted at the clinic at a time convenient to the participants and this was either after working hours and over the weekends. The Principal Investigator (LC) is a female Public Health Specialist and received training in research methods which included both qualitative research methods and analysis as part of her Master of Public Health training. The researcher introduced herself as a researcher studying towards a Master of Public Health degree and that she is also a registered nurse/midwife with one of the private clinics. The PI acknowledged her position at the facility so that the participants were aware of that. The PI reiterated to the participants that their decision against joining the study will not affect their regular medical services at the facility and that this study was not commissioned by the facility or its owners. The PI reflected on her interests in improving retention of patients on ART. The PI reflected on her interests in improving retention of patients on ART. There were no non-participants present during the interviews. The broad questions that guided the interviews were as follows:

1. Explain to me in detail the challenges in taking ARVs?
2. Explain to me in detail the factors that enable adherence to ARVs?

We probed further after the initial broad questions to capture more depth on the subject as per topic guide (Additional file 1). Interviews were deliberated in both English and Chichewa as per participants’ preference. All interviews were captured using a digital audio recorder and each recorded interview was assigned a unique identification number. Audio records and completed transcripts were stored on a password-protected computer. The researcher and research assistants collected field notes while conducting the interviews. We ensured quality by summarizing our findings at the end of the each interview as a form of member check [29, 30]. We probed more for clarity [31] and to ascertain extensive coverage of the issues under discussion and also conducted the study in the participants preferred language. Each interview lasted for 30–45 min. Data collection stopped when we reached saturation point which was realized when no new information emerged information from the interviews.

### Data analysis

Data analysis was manually performed using thematic analysis as outlined by Braun and Clarke [32] and commenced during data collection period. All audio recordings were transcribed verbatim and simultaneously translated if the interview was not done in English while interviews conducted in English were transcribed immediately. Codes were deductively [30] realized from the objectives and inductively [30] realized from the data. Researchers (LC and ALNM) listened to the audios and read the transcripts several times to familiarize with the data. Initial codes were generated by LC by and ALNM by re-reading the transcripts followed by open coding of each transcript. The initial transcript was coded by two qualitative researchers (LC and ALNM) and codes were compared and areas that differed were discussed between the two researchers until a consensus was reached regarding the most plausible code for that aspect [32]. A coding guide was the output of the consensus and then all transcripts were coded by LC. We searched for themes from the codes by collating all similar and recurrent codes under an overarching theme. We examined each code for further subcategories [32]. We reviewed the themes and this resulted in maintaining, combining, separating or discarding themes as necessary [32] and the decision to change was dependent on the richness and breadth of the proposed theme to accommodate other sub themes. For instance, we combined the findings on time when ARVs are taken under a sub theme of forgetfulness because they relayed the same message. We did this to ensure that all themes presented had rich data that substantiated the theme, which resulted into discarding of themes without supporting data. We refined the themes and verified our results against the digital recordings.

## Results

### Demographic characteristics of health care workers

We interviewed 6 ART providers and of these, two were clinical officers, two were ART nurses and the other two were ART clerks. Their ages ranged from 33 to 40 with a median of 36.5 and 3 were males.

### Demographic characteristics of study participants

We interviewed a total of 24 ART clients. Of the 24 clients, 12 had defaulted from ART, and six were male participants. The other 12 participants were compliant to their ART and six were male participants. The age range of the participants was from 30 to 50 years with a median of 38 years. Participants were either employed by the government or a private entity or were self-empoyed. Twenty participants were on first line regimen comprising Lamivudine, Tenofovir and Efavirenz (5A regimen) while the other four were on second line treatment comprising Tenofovir, Lamivudine plus Atazanavir (7A regimen). All participants initiated ART treatment in the period from 1st January 2012 to 31st December 2015 and all resided in urban areas (Table 1).

**Table 1:** Demographic characteristics of study participants *N* = 24

Variable	Defaulter (n)	non_defaulter (n)
gender		
Male	6	6
Female	6	6
participant age		
20–30	2	2
31–50	10	9
> 50	0	1
Education Level		
Secondary	2	8
University	10	4
Employment Status		
Unemployed	0	1
self employed	3	2
employed by government	2	5
employed in private	7	4
residence		
Rural	0	0
Urban	12	12
treatment		
first line	9	11
second line	3	1
Disclosure		
Yes	4	12
No	8	0
marital status		
Single	3	2
Married	9	10
Divorced	0	0
Widow	0	0
Duration on ART		
less than 2 yrs	0	1
2–4 yrs	9	9
> 4 yrs	3	2

#### Factors that influence adherence to Antiretrovirals

The factors that influence adherence and retention in ART care are at individual and health system factors. The personal factors that influence adherence and retention to ART include forgetfulness and better perception of one’s health.

#### Patient level factors

##### Forgetfulness

Clients and health workers stated that most clients miss their doses because they forget to take the ARVs and that is compounded if a client has no-one to remind them about the drugs. It was observed that presence or absence of support is an important factor in adherence to ARV drugs. Most participants hoped that they would receive support and encouragement from family and friends, considering that ART is for life-time duration. Unfortunately, many received no support from family or friends after disclosure. One participant explained as below:

*“My wife knows my status but does not support me because she is HIV-negative herself. Sometimes she tells me that she is tired of looking after me because I am HIV-positive. In such cases, sometimes I don’t even take the drugs.”* (Male participant, non-adherent)

Participants further reported that in instances when clients run out of their ARVs and have no one to remind them about refilling their drugs, some opted to default especially if they had missed taking treatment for a long period of time.

Participants also attributed forgetfulness to intake of alcohol. One participant concluded that:

“*After drinking alcohol, I could not remember to take the drugs since I will go home very late and no one would remind me to take the ARVs.”* (Male participant, non-adherent)

Health workers noted that an individual’s behavior significantly contributes to rates of defaulting among adults who are on ART. A health care worker stated:

*“Those clients who are addicted to smoking and alcohol, tend to forget to take the ARVs.”* (Health Provider, Clinician)

##### Better perception of one’s health

Participants in the study reported that defaulting from treatment also occurs after experiencing a positive change in one’s health especially when one is virally suppressed. This is illustrated below:

*“Before I started (on ARVs), I was often sick but when I started ARVs, I immediately got well and was fit just like those who are not on ARVs and I thought I was cured so I did not see any reason to continue taking ARVs.”* ( Female participant, non-adherent)

Health care workers also collaborated that a better perception of health affects adherence to ARVs. One health care worker explained as follows:

*“I don’t understand some of these clients, because when we are initiating on treatment we normally give them information so that they should understand why they are taking ARVs every day, but once they get well, most of them they default from treatment thinking that they are cured especially after you tell them that their viral load is undetectable.”* (Health Provider, ART Clerk)

##### Denial of an HIV infected status

Some clients are non-adherent to treatment as a result of failure to accept their HIV-positive status. This is compounded with inadequate knowledge about the consequences of defaulting from treatment. The quotes below illustrates this:

*“Honestly I did not accept and I still ask my God, why me? As of now I cannot do anything because I know I will die just now, so the best option I should do is that I should be drinking alcohol and smoking.”* (Male participant, non-adherent)

*“I never expected this in my life, I never had girlfriends apart from my wife, so I wonder where this disease you call HIV is coming from.”* (Male participant, non-adherent)

Health workers also collaborated that denial of one’s HIV-infected status explains some clients’ behavior on defaulting from ARVs:

*“What I have observed in these clients is that they do not want to accept their status after testing positive. So if they have not accepted their status, it is difficult to take the ARVs hence any day one may decide to default from treatment.”* (Health Provider, Nurse)

##### Fear of disclosure of an HIV-infected status

Fear of disclosure of one’s status was the most common reason for defaulting from ARVs. Most clients do not want their relatives or partner status to learn their positive status for fear of being stigmatized because of their positive status. In such cases, there is no one to remind them when they miss the drugs.

“*I did not want to tell anyone about my HIV status. I did not want to be a laughing stock that am HIV-positive’.* (Male participant, non-adherent)

*“Ah why should I tell people? … Eh I cannot do that I don’t want to be exposed.”* (Male participant, non-adherent)

A health care worker collaborated on the same and reported as follows:

*“Some people really default from treatment just because they don’t want to tell their relatives, even a partner just because they are afraid that they will be laughed at if their loved ones come to realize that they are HIV-positive.”* (KII 002)

##### ARVs side effects

Some participants expressed concern that side effects from ART initiation such as vomiting, skin rashes, and jaundice contributed to defaulting from treatment. Participants narrated that although they were informed that they will get better, they developed severe side effects which resulted in defaulting from their ARVS to avert the side effects. One participant explained:

*“I don’t know what happens to other people when they start ART, they become sicker than before so they would rather stop and get better than continuing with ART.”* (Female participant adherent)

Another participant explained:

*“I was indeed sick but when I started ARVs my condition got worse. I was vomiting every day and also experienced dizziness plus headache. So when I went back to the clinic, I was told that I would get better and yet my condition got worse so I decided to stop so that at least I should be relieved as I was before.”* (Female participant, non-adherent)

##### ARV fatigue

Participants stated that most clients were felt of taking ARVs on a daily basis and also that it would be a life-long treatment. At times, participants would decide to miss the dose deliberately for temporal rest but later decide to default from treatment.

*“Honestly I was tired of taking drugs every day. It was just too much, I wanted to rest a bit.”* (Female participant, non-adherent)

“*Many clients are defaulting from treatment because they are tired of taking ARVs daily and also with the fact that they will take the ARVs for the rest of their life.” (*Health Provider, Clinician)

Usage of alternative medicines also influenced defaulting among clients. Participants referred to some traditional medicines that work better but are not taken on a daily basis such as herbal medicines. The fact that one could initiate and stop herbal medicines at any point when they felt better was an attraction to alternative treatment unlike ARVs that have to be taken daily for a lifetime.

*“Ah I don’t see any reason for taking ARVs, the drugs are made from herbs so it is good for me to take herbs for example “Terasi” (a herbal mixture believed to boost one’s immunity) any time I feel like rather than these ARVs… taking these drugs daily give me a tough time”.* (Female participant, non-adherent)

### Health system level factors

#### Human resources factors

##### Poor relationship between health care workers and participants

Participants stated that poor relationships between health care workers and ART clients also contributed to participants defaulting from ART.

*“Sometimes some health workers do not interact well with clients, they shout…so based on how they were raised up and the respect they receive in society, some clients decide to stop rather than go to the hospital for a refill and get embarrassed at the facility.”* (Male participant, adherent)

##### Shortage of ART trained health care workers

Both health workers and clients reported that shortages of ART providers led to clients experiencing long waiting time which could encourage defaulting from ART care. The shortage of staff results in sub-optimal counseling on the ARVs resulting into patients obtaining inadequate knowledge about the benefits of ARVs. This may translate into patients defaulting from treatment as explained below:

*“Health workers too are contributing to default from treatment, sometimes due to staff shortage. With many clients waiting to be initiated on ART, we sometimes involve untrained nurses to assist in counseling so that we clear the queue. It’s obvious the untrained staff may not provide adequate information thereby clients can easily default from treatment.”* (Health Provider, Clinician)

#### Financial and geographical accessibility of ARVs

Financial and geographical accessibility were also linked to ART default. The lack of finances coupled with the distance that the clients had to cover to reach the privately owned facilities negatively affected adherence to ARVs. Participants reported of other ART clients who defaulted from treatment because of economic demands associated with transport and time spent to refill their drugs.

*“Money is a problem, even businesses are not going on well, so for someone who is not working but doing business only cannot manage every month to come for refill. Where is he/she going to find money for transport? Later they will start missing the appointments and then in the end will completely stop.”* (Male participant, adherent)

Participants also highlighted that long distances contribute to defaulting from ARVs.

*“Some clients stay far away from the facility and cannot manage to come monthly for a refill because it is not always the case that they have money every day. For those who stay a bit close to the hospital, they can walk to the clinic for a refill when they don’t have transport money.”* (Female participant, adherent)

#### Facilitators to ART adherence

Facilitators to ART adherence included individual and health system-related factors. At individual level, the significant factor was psychological support while at health system they included follow up visits after missed visits, positive relationships with health workers and adequate Information, education and counseling.

#### Individual level factors

##### Psychological support

It was noted that psychological support from health workers, family, and community members played a positive role in maintaining adherence among those in ART care. Psychological support facilitated acceptance of one’s positive status, helped eliminate stigma, and created a platform where a client was encouraged and reminded about taking drugs.

*“It is not easy to be told that you are HIV-positive and you accept immediately. It is a shock so these people normally go through denial process before they accept, so when they get support from the family, health workers, even community, they get encouraged. As a result, they have less chances of defaulting from ART.”* (Health Provider, Nurse)

### Health systems factors

#### Follow up visits after missed visits

It was narrated that for clients who miss two consecutive appointment dates, private facilities should trace them to their homes to explore on the missed appointments. Follow-up visits would provide a platform for identification of problems leading to non-adherence and offer room for further counseling sessions.

*“When health workers observe that they are some clients missing appointments for two months, they have to go in to their houses since when starting treatment we are asked our physical addresses. So if they visit them at their homes they need to find out why they are not coming for refill, and should help them. This can help to reduce many clients to default from treatment.”* (Male participant, adherent)

Health workers argued that:

*“The main problem is with us the ART providers, we see that clients are not coming for refill for two consecutive months and we just observe instead of taking action and following it up and in order to find out the problem and address it.”* (Health Provider, ART Clerk)

#### Positive relationship with health workers

A good relationship with health workers enhances adherence to ART treatment. This is expressed below:

*“When we were told that we are HIV-positive honestly, we felt like we will die tomorrow, so when we go for refill of drugs we are already stressed up….if they can welcome and interact well with us, we can open up and get assisted well… do you think one can then decide to default from treatment?”* (Male participant, adherent)

#### Adequate information, education and counseling

Health workers reported on the need for intensive counseling especially for newly initiated individuals including both benefits and consequences of ART care and default by trained health workers. Comprehensive counseling promotes clients understanding of their treatment and may reduce the non-adherence rate from ARVs.

*“To say the truth, some ART providers are not well trained as a result may not give adequate information to clients leading to clients not understanding…why they are taking ARVs including the benefits…or even for how long will they take ARVs for. So once the client is blank and does not know all these, they can easily decide to stop treatment anytime.”* (Health Provider, ART Clerk)

Participants also mentioned the importance of sensitizing clients about possible side effects upon commencement of treatment as a retainer in ART care.

*“When clients are initiated on ART, they sometimes develop side effects and yet they were not told during counseling process. When they go back to the clinic for assistance, health workers may not attend to them properly and at times they are only told to go back and they will be fine and yet that client is very sick. When they go back home, they may decide to default since they were not assisted well.”* (Female participant, adherent)

## Discussion

Factors influencing defaulting from ART care include both individual and health system related dynamics. Individual barriers include forgetfulness, better perception of one’s health, denial of an HIV-infected status, fear of disclosure of one’s positive status, side effects and ART fatigue. Health system dynamics include human resource factors and accessibility of services. Factors affecting retention into ART care include psychological support, follow up visits after a missed visit, supportive relationships and adequate information education and counseling after a positive diagnosis.

Our findings on forgetting to take ARVs secondary to use of substances is similar to previous studies that attributed defaulting to forgetfulness due to alcohol and drugs use [1, 3, 6, 8, 33]. Non-adherence to ARVs in the context of alcohol use is influenced by a desire to keep one’s HIV status private, thereby omitting taking ARVs whilst taking alcohol [33]. Additionally, the fear of side-effects resulting from combined toxicity of drugs and alcohol [33] results in defaulting. Conversely, abstinence from alcohol is associated with adherence to ARVs [34]. We suggest inclusion and exploration of social habits as an ongoing process with ART care and adoption of the Cutting down, Annoyance by criticism, Guilty feeling and Eye-openers (CAGE) questionnaire [35] with prompts for referral. To realize such linkage to care Malawi needs to establish or map out alcoholic support groups introduced or integrated within ART care for participant referral. In South Africa, health care workers expressed inadequate training and knowledge on ARVs and alcohol intake and recommended capacity strengthening in that aspect [36] which may also be applicable to Malawi. Counsellors could lead in alcohol related counseling as health workers have been deemed to be nonjudgmental on usage of alcohol while on ARVs [36].

Experiences with drugs may either prompt clients to take ARVs timely and correctly or abandon the regimen. Specifically in our study, a better perception of health [1, 3, 6, 13] and a bad experience with side effects influenced defaulting from treatment. Additionally, a systematic review reported that patients with compromised immunity with repeated illnesses which interfered with clinic appointments, missed their ART refill appointments than those that were stable [3, 37]. Furthermore, experiencing side effects from drugs remains a common reason to defaulting from ART treatment [3, 9, 13, 37]. Other participants default from treatment after initiating alternative medicines from biomedical concoctions, traditional or herbal medicine or locally promoted concoctions [38, 39]. Stakeholders in alternative medicines need to be involved in ART care to promote adherence amongst clients. A mapping exercise of the available providers for alternative medicines would ease targeting of their inclusion.

The fear of disclosing one’s HIV infected status was the most common reason reported by most clients in this study and remains similar to previous findings [38]. The findings remain consistent with a study in Malawi that reported that most clients deny their HIV status resulting in a poor understanding of the benefits of ART [13]. The same study reported that lack of adequate information affected daily intake of ARVs [13]. A study from Uganda, Botswana and Tanzania reported that consequences of non-disclosure among patients on ART is the covert taking of medications which leads to either delayed or missed medications that lead to defaulting from ART [3]. Contrary, early and full disclosure of one’s HIV infected status has been associated with improved adherence to one’s treatment [40]. We propose promotion of facilitated mutual disclosure [41] of one’s HIV status and also strengthening interventions that overcome stigma and discrimination in the country. Integration of mental health services within ART clinics would assist the clients in navigating through acceptance of their results and this could be in the form of a friendship bench [36]. Furthermore, we recommend a hotline [33] to enable participants talk with a mental health expert on how to navigate HIV related aspects.

Defaulting from treatment secondary to ARV fatigue resonates with previous studies that stated that majority of clients defaulted from treatment because of fatigue [4, 6, 36, 37, 38]. Loeliger [42] argued that clients would delay initiation of ARVs because of the life-treatment duration associated with ARVs. The potential of having simpler regimens that are taken once a month or lesser in a year may be a likely solution to this specific barrier [37]. Additionally, adoption of expert clients [29] in private facilities may assist with counseling and peer support for participants that are struggling with compliance. The Malawi Government ART guidelines insists on having a guardian for the ART clients, and the guardian is counseled alongside the client on the regimen and compliance, and this could be adopted in the private setting for willing participants.

Our study revealed that some clients defaulted from ART treatment due to poor relationship with health workers [1, 3, 6]. The notion of patient centeredness [33] which is a premise for quality health services needs to be intensified in provision of ART services. Patient centeredness promotes active participant involvement and considers them as experts in their care [43]. This notion is likely to bring the patients’ views about their health to the knowledge of the health care workers. Consequent to staff inadequacy, our study revealed that participants received sub optimal counseling on their ARVs treatment. Similarly, studies in several African countries reported that health facilities are faced with inadequacy of human resource against handling the increased numbers of clients [3, 6, 14] which results in sub optimal counseling about the disease and the benefits of ART thereby inadvertently affecting retention [3, 9].

The financial burden imposed on the clients as expressed in this study is congruent with findings from studies in Nepal and other African countries that reported transportation costs acts as a barrier in accessing treatment [3, 6, 9, 39] and regarded the process of refilling their ARVs economically demanding [11]. Related to financial accessibility, our study showed that geographical accessibility led to defaulting from treatment and builds on findings from previous studies that reported that patients who resided in rural areas and far from the health facility defaulted from ART treatment because of the long distance [3, 6, 9]. However, most of the clients served by the private clinics are not from the rural areas which raises the possibility that the geographical accessibility observed in this study is closely linked to the financial accessibility since there are fewer private facilities available as compared to the government-owned facilities. Private health facilities could orient clients to alternative free ART centers closer to where one resides in the event that one is financially constrained.

Psychosocial support enhances ART adherence [44] and the support could be from health workers, family, and community members [39, 45]. Clients who received support reported to have felt accepted, non-stigmatized, non-discriminated and were encouraged which promoted adherence to ARVs [46–48]. According to this study, clients who miss two consecutive appointment dates need be traced at their homes to establish the reasons behind missed appointments. Outreach clinics which were rolled out in East Africa to monitor and provide ARVs for the clients and also re-link those who defaulted back to treatment [36, 45] improved follow up of clients. Private facilities may liaise with the other private clinics within the vicinity of their clients and be able to refer or transfer them to a facility nearest to their place.

Good relationships with health care providers promotes ART adherence among clients [45, 49–51]. They also build good rapport which creates a platform for sharing information and enhances counseling for the provider and the participants. The Malawi Business Coalition against AIDS, which is an overarching body overseeing HIV Service provision in the private sector, ought to ensure that the providers in the private setting are trained in customer care for the provision of quality HIV services. Other support that may promote adherence would be on management of alcohol use through the assessment and incorporation of other services addressing alcohol use [52]. In other settings traditional healers have been accepted as community partners who may ensure adherence to ART [53]. A good integration and recognition of suppliers of alternative medicines and asking them to ensure and emphasize on compliance may assist adherence among selection of participants that visit them.

Our finding on the provision of adequate information, education and counseling builds upon findings from a previous studies where clients adhere to ART treatment through intensive education and counseling that focuses on direct patient feedback, patient problem solving, self-monitoring and patient education [46, 47] which are also premises of patient centeredness [43].

### Strengths and limitations

Although our study provides important insights for provision of HIV services in private settings in Malawi, it had some limitations. This study is limited to the selected private clinics where we sampled from and because private clinics conduct HIV services differently from government facilities, the results may only be applicable in the settings it was done. Identification of participants was a challenge as it required more time to reach them. This study is one of the few that has sampled from private facilities in Malawi as opposed to most studies that take place in the ART settings within the Government owned clinics. Although the principal researcher worked at one of the facilities, the study participants were encouraged to freely express their views on the matter and were assured that what they will share will not affect their regular access to health services but would be used to improve their HIV services at the facility. We were not able to take the findings back to the participants however we summarized the key points after each interview and participants verified our findings. We also acknowledge the limitation of not calculating a Kappa to ascertain agreement between coders, however we continuously discussed the results and the codes and themes as they emerged to ensure that the data was credible.

## Conclusion

In this study, the main reason for defaulting from ARVs was fear of disclosing an HIV status to avert potential stigma and discrimination. In implementing ART clinics due consideration and strategies need to be adopted to ensure that privacy and confidentiality is preserved. Although adoption of all the key Malawi Implementing strategies like expert clients and a guardian may optimize retention in care, there is need for prior analysis of how those may lead to unintended disclosure which inadvertently affects adherence. Furthermore, private facilities should orient their clients to the public facilities within the catchment area so that clients have an option for alternative access to HIV care in the event of financial constraints. Further research should also look at development and acceptability of referral networks between private and public health facilities to optimize adherence to ARVs among clients.

## Supplementary information

**Additional file 1. **Codebook.

## Data Availability

The datasets used are available from the corresponding author on request.

## Abbreviations

AIDS: Acquired Immunodeficiency Syndrome
ART: Antiretroviral Therapy
ARVs: Antiretrovirals
CAGE: Cutting down, Annoyance by criticism, Guilty Feelings and Eye openers
HIV: Human Immunodeficiency Virus
PI: Principal Investigator

## References

[1] Kranzer K, Lewis JJ, Ford N, Zeinecker J, Orrell C, Lawn SD, Bekker LG, Wood R (2010). Treatment interruption in a primary care antiretroviral therapy programme in South Africa: cohort analysis of trends and risk factors. J Acquir Immune Defic Syndr.

[2] Ministry of Health: Department of HIV & AIDS. Government of Malawi: Lilongwe: Statistics for ART in Malawi [home page internet].2018[updated 2018; cited 2018 Dec 3]. Available from; http://www.hiv.health.gov.mw/. Accessed 3 Dec 2018.

[3] Wasti Sharada P., van Teijlingen Edwin, Simkhada Padam, Randall Julian, Baxter Susan, Kirkpatrick Pamela, GC Vijay S. (2011). Factors influencing adherence to antiretroviral treatment in Asian developing countries: a systematic review. Tropical Medicine & International Health.

[4] Shet A, De Costa A, Heylen E, Shastri S, Chandy S, Ekstrand M. High rates of adherence and treatment success in a public and public-private HIV clinic in India: potential benefits of standardized national care delivery systems. BMC health services research[Internet]. 2011 Oct 17 [cited 2016 Oct];11(1):277.doi: 10.1186/1472-6963-11-277 10.1186/1472-6963-11-277 PMC3204232 22004573

[5] Forsythe SS (1998). The affordability of antiretroviral therapy in developing countries: what policymakers need to know. AIDS.

[6] Hardon A, Davey S, Gerrits T, Hodgkin C, Irunde H, Kgatlwane J, et al. From access to adherence: the challenges of antiretroviral treatment. World Health Organization 2006. Available from: http://www.live.who.int/entity/medicines/publications/challenges_arvtreatment15Aug2006.pdf.

[7] Asefa T, Taha M, Dejene T, Dube L (2013). Determinants of defaulting from antiretroviral therapy treatment in Nekemte Hospital, Eastern Wollega Zone, Western Ethiopia. Public Health Res.

[8] Jones D, Cook R, Cecchini D, Sued O, Bofill L, Weiss S (2015). Examining adherence among challenging patients in public and private HIV care in Argentina. AIDS Behav.

[9] Shubber Zara, Mills Edward J., Nachega Jean B., Vreeman Rachel, Freitas Marcelo, Bock Peter, Nsanzimana Sabin, Penazzato Martina, Appolo Tsitsi, Doherty Meg, Ford Nathan (2016). Patient-Reported Barriers to Adherence to Antiretroviral Therapy: A Systematic Review and Meta-Analysis. PLOS Medicine.

[10] Sasaki Y, Kakimoto K, Dube C, Sikazwe I, Moyo C, Syakantu G (2012). Adherence to antiretroviral therapy (ART) during the early months of treatment in rural Zambia: influence of demographic characteristics and social surroundings of patients. Ann Clin Microbiol Antimicrob.

[11] Miller CM, Ketlhapile M, Rybasack-Smith H, Rosen S (2010). Why are antiretroviral treatment patients lost to follow-up? A qualitative study from South Africa. Trop Med Int Health.

[12] Wasti SP, Simkhada P, Randall J, Freeman JV, van Teijlingen E (2012). Factors Influencing Adherence to Antiretroviral Treatment in Nepal: A Mixed-Methods Study. PLOS ONE.

[13] McGuire Megan, Munyenyembe Tamika, Szumilin Elisabeth, Heinzelmann Annette, Le Paih Mickael, Bouithy Nenette, Pujades-Rodríguez Mar (2010). Vital status of pre-ART and ART patients defaulting from care in rural Malawi. Tropical Medicine & International Health.

[14] Wasti SP, Simkhada P, Teijlingen ER. Antiretroviral treatment programmes in Nepal: Problems and barriers. Kathmandu Univ Med J KUMJ [Internet]. 2009 Sep [cited 2017 Oct]; 7(27):306–14. Available from: http://eprints.bournemouth.ac.uk/11763 10.3126/kumj.v7i3.2743 20071882

[15] Musheke M, Bond V, Merten S (2012). Individual and contextual factors influencing patient attrition from antiretroviral therapy care in an urban community of Lusaka, Zambia. J Int AIDS Soc.

[16] Holtzman CW, Shea JA, Glanz K, Jacobs LM, Gross R, Hines J (2015). Mapping Patient–Identified Barriers and Facilitators to Retention in HIV Care and Antiretroviral Therapy Adherence to Andersen’s Behavioral Model. AIDS Care.

[17] Merten S, Kenter E, McKenzie O, Musheke M, Ntalasha H, Martin-Hilber A (2010). Patient-reported barriers and drivers of adherence to antiretrovirals in sub-Saharan Africa: A meta-ethnography. Trop Med Int Health.

[18] Sulzbach S., De S., Wang W. (2011). The private sector role in HIV/AIDS in the context of an expanded global response: expenditure trends in five sub-Saharan African countries. Health Policy and Planning.

[19] Nakhimovsky S, Callahan S, Gatome-Munyua A. The role of the privates sector in the HIV response of four countries. Shops plus 2014. Available from;https://www.shopsplusproject.org/sites/default/files/resources/The%20role%20of%20the%20private%20sector%20in%20the%20HIV%20response%20of%20four%20countries.pdf. Accessed 19 July 2018.

[20] Malawi Private Health Sector Mapping Report. USAID. 2013 Available: https://www.shopsplusproject.org/sites/default/files/resources/Malawi%20Provider%20Mapping%20Report.pdf. Accessed 19 July 2018.

[21] Private Facility 1. ART register book 1.[unpublished] Malawi: 2012.

[22] Private Facility 2 ART register book 1.[unpublished] Malawi: 2012.

[23] Malawi Guidelines for Clinical Management of HIV in Children and Adults Dept for HIV and AIDS [internet] 2016 [cited 2017 October 23]. Available from: www.hiv.health.gov.mw. Accessed 23 Oct 2017.

[24] Lambert V, Lambert C, Qualitative descriptive research: an acceptable design. Pacific Rim International Journal of Nursing Research, vol. 16, pp. 255–256, 2013, http://antispam.kmutt.ac.th/index.php/PRIJNR/article/download/5805/5064. Accessed 1 Nov 2017.

[25] Mason J. Qualitative researching. 2nd ed. London ; Thousand Oaks, Calif: Sage Publications [Internet]. 2002 [cited 2017 Nov 12]. Available from: www.sxf.uevora.pt/wp-content/uploads/2013/03/Mason_2002.pdf. Accessed 12 Nov 2017.

[26] Gill P., Stewart K., Treasure E., Chadwick B. (2008). Methods of data collection in qualitative research: interviews and focus groups. British Dental Journal.

[27] Sandelowski M (1995). Sample Size in Qualitative research. Res Nurs Health.

[28] Ritchie J, Lewis J (2003). Qualitative Research Practice, A Guide for Social Science Students and Researchers.

[29] Leung L.. Validity, reliability, and generalizability in qualitative research. J Fam Med Prim Care[homepage internet]. 2014 [updated 2015 Sep; cited 2017 Nov 14]; 4(3):324–7.Available from: www.jfmpc.com/article.asp?issn=2249-4863;year=2015;volume=4 10.4103/2249-4863.161306 PMC4535087 26288766

[30] Hennink M, Hutter I (2011). A. B: Qualitative Research Methods.

[31] Clark JP, Godlee F, Jefferson T (2003). How to peer review a qualitative manuscript. Peer Review in Health Sciences, 2nd edn.

[32] Braun V, Clarke V (2006). Using thematic analysis in psychology. Qual Res Psychol.

[33] Scholl Isabelle, Zill Jördis M., Härter Martin, Dirmaier Jörg (2014). An Integrative Model of Patient-Centeredness – A Systematic Review and Concept Analysis. PLoS ONE.

[34] Kessler Jason, Nucifora Kimberly, Li Lingfeng, Uhler Lauren, Braithwaite Scott (2015). Impact and Cost-Effectiveness of Hypothetical Strategies to Enhance Retention in Care within HIV Treatment Programs in East Africa. Value in Health.

[35] Ewing John A. (1984). Detecting Alcoholism. JAMA.

[36] Schneider M. Chersich M. Temmerman M. and Parry C.D, Addressing the intersection between alcohol consumption and antiretroviral treatment: needs assessment and design of interventions for primary health care workers, the Western Cape, South Africa. Globalization and Health[ internet] 2016 12:65[ cited 2018 July 26]; DOI 10.1186/s12992-016-0201-9 10.1186/s12992-016-0201-9 PMC5080779 27784302

[37] Loeliger Kelsey B., Niccolai Linda M., Mtungwa Lillian N., Moll Anthony, Shenoi Sheela V. (2016). “I Have to Push Him with a Wheelbarrow to the Clinic”: Community Health Workers' Roles, Needs, and Strategies to Improve HIV Care in Rural South Africa. AIDS Patient Care and STDs.

[38] Azia IN, Mukumbang FC, Van Wyk B (2016). Barriers to adherence to antiretroviral treatment in a regional hospital in Vredenburg, Western Cape, South Africa. S Afr J HIV Med.

[39] Simon M. Kang’ethe and Thanduxolo Nomngcoyiya Exploring Underpinnings Weighing Down the Phenomenon of Adherence to Anti-Retroviral Drugs (ARVs) among the People Living With HIV/AIDS (PLWHA) in South Africa and Botswana:A Literature Review. Kamla-Raj [internet] 2015 [cited 2018 July 22]; 50(3): 237–243 (2015.

[40] Musumari PM, Wouters E, Kayembe PK, Kiumbu Nzita M, Mbikayi SM, Suguimoto SP, et al. Food Insecurity Is Associated with Increased Risk of Non-Adherence to Antiretroviral Therapy among HIV-Infected Adults in the Democratic Republic of Congo: A Cross-Sectional Study. Sued O, editor. PLoS ONE [Internet] .2014 Jan 15 [cited 2017 Oct 12]; 9(1):e85327. 10.1371/journal.pone.0085327 PMC3893174 24454841

[41] Cluver Lucie D., Hodes Rebecca J., Toska Elona, Kidia Khameer K., Orkin F. Mark, Sherr Lorraine, Meinck Franziska (2015). ‘HIV is like a tsotsi. ARVs are your guns’. AIDS.

[42] Loeliger KB, Niccolai LM, Mtungwa LN, Moll A, Shenoi SV (2016). Antiretroviral therapy initiation and adherence in rural South Africa: community health workers' perspectives on barriers and facilitators. AIDS Care.

[43] De Man J, Mayega RW, Sarkar N, Waweru E, Leys M, Van Olmen J (2016). Patient-Centered Care and People-Centered Health Systems in Sub-Saharan Africa: Why So Little of Something So Badly Needed?. Int J Pers Cent Med [Internet].

[44] Bijker Rimke, Jiamsakul Awachana, Kityo Cissy, Kiertiburanakul Sasisopin, Siwale Margaret, Phanuphak Praphan, Akanmu Sulaimon, Chaiwarith Romanee, Wit Ferdinand W, Sim Benedict LH, Boender Tamara Sonia, Ditangco Rossana, Rinke De Wit Tobias F, Sohn Annette H, Hamers Raph L (2017). Adherence to antiretroviral therapy for HIV in sub-Saharan Africa and Asia: a comparative analysis of two regional cohorts. Journal of the International AIDS Society.

[45] Chen W-T, Wantland D, Reid P, Corless IB, Eller LS, Iipinge S, et al. Engagement with Health Care Providers Affects Self- Efficacy, Self-Esteem, Medication Adherence and Quality of Life in People Living with HIV. J AIDS Clin Res [Internet]. 2013 Nov 1[cited 2017 Nov 12]; 4(11):256. doi: 10.4172/2155-6113.1000256 10.4172/2155-6113.1000256 PMC3932545 24575329

[46] Sahay Seema, Dhayarkar Sampada, Reddy KSrikanth (2011). Optimizing adherence to antiretroviral therapy. The Indian Journal of Medical Research.

[47] Kalichman Seth C., Kalichman Moira O., Cherry Chauncey, Eaton Lisa A., Cruess Dean, Schinazi Raymond F. (2016). Randomized Factorial Trial of Phone-Delivered Support Counseling and Daily Text Message Reminders for HIV Treatment Adherence. JAIDS Journal of Acquired Immune Deficiency Syndromes.

[48] Wang Kerong, Chen Wei-Ti, Zhang Lin, Bao MeiJuan, Zhao Hongxin, Lu Hongzhou (2016). Facilitators of and barriers to HIV self-management: Perspectives of HIV-positive women in China. Applied Nursing Research.

[49] Rackal JM, Tynan A-M, Handford CD, Rzeznikiewiz D, Agha A, Glazier R. Provider training and experience for people living with HIV/AIDS. In: Cochrane Database of Systematic Reviews. John Wiley & Sons, Ltd [Internet]. 2011 [cited 2017 Oct 8]. Availablefrom:http://onlinelibrary.wiley.com/doi/10.1002/14651858.CD003938.pub2/abstract 10.1002/14651858.CD003938.pub2 21678344

[50] Corless Inge B., Wantland Dean, Kirksey Kenn M., Nicholas Patrice K., Human Sarie, Arudo John, Rosa Maria, Cuca Yvette, Willard Sue, Hamilton Mary Jane, Portillo Carmen, Sefcik Elizabeth, Robinson Linda, Bain Cathy, Moezzi Shanaz, Maryland Mary, Huang Emily, Holzemer William L. (2012). Exploring the Contribution of General Self-Efficacy to the Use of Self-Care Symptom Management Strategies by People Living with HIV Infection. AIDS Patient Care and STDs.

[51] Lester RT, Mills EJ, Kariri A, Ritvo P, Chung M, Jack W, Habyarimana J, Karanja S, Barasa S, Nguti R, Estambale B. The HAART cell phone adherence trial (WelTel Kenya1): a randomized controlled trial protocol Trials [internet]. 2009 Sep 22[cited 2017 Oct] ; 10(1):87. 10.1186/1745-6215-10-87 PMC2760542 19772596

[52] Jeffrey H. Samet, Nicholas J. Horton, Seville M, Kenneth A. Freedberg, and Palepu A. Alcohol Consumption and Antiretroviral Adherence Among HIV-Infected Persons With Alcohol Problems Alcoholism: Clinical and Experiment. [internet] 2006[cited 2018 July 20]; Available: http://login.research4life.org/tacsgr1doi_org/10.1097/01.ALC.0000122103.74491.78 10.1097/01.alc.0000122103.74491.78 15100608

[53] Audet CM, Ngobeni S, Wagner R. Traditional healer Treatment of HIV persists in the era of ART: a mixed methods study from rural South Africa. BMC Complementary and Alternative medicine BMC series [internet] 2017 17:434 [cited 2018 July 26; 10.1186/s12906-017-1934-6 PMC5577748 28854905

