# Supplementary material for: Factors influencing adherence to antiretroviral treatment among adults accessing care from private health facilities in Malawi
Source: BMC Public Health. 2019 Oct 28;19:1382. doi: 10.1186/s12889-019-7768-z (PMC6816213; doi:10.1186/s12889-019-7768-z)
Supplement: Supplementary file 1 — Additional file 1. Codebook. [file 12889_2019_7768_MOESM1_ESM.docx]

**Codebook - (only shows the section for this paper)**

| **Nodes** | **Sub-nodes** | **Classification Level** |
| --- | --- | --- |
| Barriers | - Discussions anything that would hinder compliance to ART and could range from: Financial, ARVs side effects , Healthcare worker issues, health system Factors - Stigma - Non disclosure of an HIV status ,Religious beliefs and personal values - Community Factors - Perceptions of one’s health - Denial of an HIV infected status | Individual or Patient Level  Health System Level |
| Facilitators | - Discussion on anything that enables one to remain compliant to ARVs - Support one receives to be retained in care - Discussion on anything others have said that it is used to be retained incur.It could also mean the policies available, systems under which services are delivered. - Drug Factors such as regimen, absence of side effects - Perceived benefits like viral suppression and better health | Individual or Patient Level  Health System Level |
